# Pre-existing *Helicobacter pylori* serum IgG enhances the vibriocidal antibody response to CVD 103-HgR live oral cholera vaccine in Malian adults

**DOI:** 10.1038/s41598-020-71754-9

**Published:** 2020-10-09

**Authors:** Khitam Muhsen, Samba O. Sow, Milagritos D. Tapia, Fadima C. Haidara, Mardi Reymann, Valeria Asato, Wilbur H. Chen, Marcela F. Pasetti, Myron M. Levine

**Affiliations:** 1 Department of Epidemiology and Preventive Medicine, School of Public Health, Sackler Faculty of Medicine, Tel Aviv University, Ramat Aviv, 6139001 Tel Aviv, Israel; 2 Centre pour le Développement des Vaccins, Bamako, Mali; 3 Center for Vaccine Development and Global Health, University of Maryland School of Medicine, Baltimore, MD USA

**Keywords:** Infection, Biomarkers, Gastroenterology, Medical research

## Abstract

Accumulating evidence indicates that persistent *Helicobacter pylori* gastric infection influences immune responses to oral enteric vaccines. We studied the association between pre-existing *H. pylori* serum IgG and serum pepsinogens levels (PGs) as markers of gastric inflammation and the immune response to single-dose live oral cholera vaccine CVD 103-HgR in Malian adults. Baseline sera obtained during a phase 2 safety/immunogenicity clinical trial of cholera vaccine CVD 103-HgR among 93 healthy Malian adults were tested for *H. pylori* IgG antibodies and PGI and PGII levels using enzyme linked immunosorbent assays. Overall 74/93 (80%) vaccine recipients were *H. pylori* IgG seropositive at baseline. Vibriocidal antibody seroconversion (≥ fourfold increase 14 days following administration of CVD 103-HgR compared to baseline) among vaccine recipients was 56%. However, vibriocidal antibody seroconversion was markedly higher among *H. pylori* seropositives than seronegatives 64% vs. 26% (*p* = 0.004); adjusted relative risk: 2.20 (95% confidence intervals 1.00–4.80; *p* = 0.049). Among *H. pylori* seropositive vaccine recipients, there were no significant associations between PGI, PGII and PGI:PGII levels and vibriocidal seroconversion. The enhanced seroconversion to oral cholera vaccine CVD 103-HgR among *H. pylori* seropositive African adults provides further evidence of the immunomodulating impact of *H. pylori* on oral vaccine immunogenicity.

## Introduction

Live attenuated oral enteric vaccines have played an important role in global public health by reducing the disease burden caused by the target pathogens. Thus, oral polio vaccine (OPV) led to eradication of transmission of wild type 2 (WPV2) (no cases since 1999) and presumed eradication of WPV3 transmission (no cases since 2012). WPV1 transmission is currently limited to just two countries, Pakistan and Afghanistan^1^. Similarly, attenuated oral rotavirus vaccines have substantially reduced gastroenteritis hospitalizations and mortality following their introduction as a routine infant vaccine^2^. Mass immunization of Chilean schoolchildren with live oral Ty21a typhoid vaccine markedly reduced the incidence of both confirmed typhoid and paratyphoid B disease^3,4^. Oral cholera vaccines have blunted the intensity of seasonal cholera epidemics in endemic areas^5^. These successes have been achieved despite the lower immunogenicity and efficacy of oral vaccines in persons living in developing countries compared to persons living in industrialized nations (reviewed in^6,7^). However, the reason(s) for the diminished immunologic response to oral live attenuated vaccines in developing countries is incompletely understood. Experience with live oral cholera vaccine CVD-103-HgR exemplifies the above phenomenon^6^. A single 5 × 10^8^ colony forming unit (CFU) dose of CVD-103-HgR elicits high titers of serum vibriocidal antibody in ~ 92–95% of US adults, and is highly protective against cholera^8–10^. However, in vaccinated children and adults living in developing countries, a higher dose (~ 5 × 10^9^ CFU) is needed to achieve similarly high vibriocidal seroconversion rates^6,11–13^. Low socioeconomic status^13^, pre-existing elevated vibriocidal titers^13^ and small bowel bacterial overgrowth were some risk factors associated with diminished vibriocidal responses^14^. Individuals with blood group O were shown to develop higher vibriocidal titers^15^, and the pre-immunization elimination of helminths was shown to significantly raise the vibriocidal antibody response in non-blood group O children^16^.

Achieving the desired immune response to a live oral vaccine requires successful passage of viable vaccine organisms through the hostile acidic gastric environment to reach the immunologic inductive sites of the gut-associated lymphoid tissue in the small intestine. *Helicobacter pylori* chronically colonizes the stomach of persons in developing countries, sometimes resulting in the development of peptic ulcers^17^ and gastric cancer in older individuals^18^ (reviewed in^19,20^). Given the high prevalence of chronic *H. pylori* infection in developing countries, the role such chronic infection in modulating the immune response to oral enteric vaccines is of particular interest. Chronic *H. pylori* infection can markedly alter gastric physiology, including gastric acid secretion^19,20^, and as a consequence can indirectly influence immune responses to a variety of antigens delivered orally, including cholera vaccine.

We previously reported a notable inverse association between baseline *H. pylori* seropositivity and vibriocidal antibody seroconversion following oral vaccination with CVD 103-HgR in Chilean children < 5 years of age^21^. In contrast, in older children 5–9 years of age, a positive association was found in relation to gastric inflammation, as measured by serum pepsinogen I (PGI)^21^. In a somewhat analogous study of a live oral typhoid vaccine conducted among U.S. adults, superior immune responses were recorded following immunization with oral attenuated *Salmonella* Typhi vaccine strain CVD 908-*htrA* in adults who were seropositive for *H. pylori* and whose serum PGs indicated more advanced gastritis^22^. Thus, the association between chronic *H. pylori* infection and the immune response to oral vaccines is complex and multifactorial, and differences according to age and socioeconomic level should be assessed.

A study of the immunogenicity of CVD 103-HgR in Malian adults offered the opportunity to examine in another geographic site (West Africa) the association of pre-existing *H. pylori* immunoglobulin G (IgG) serum antibodies and serum PGs as markers of gastric inflammation with vibriocidal antibody seroconversion following immunization of adults with this live oral cholera vaccine. Based on our previous data in different populations, our hypothesis was that *H. pylori* seropositivity would be associated with higher vibriocidal antibody seroconversion in Malian adults.

## Materials and methods

### Study design and population

We used anonymized specimens of stored sera obtained from participants in a phase II clinical trial with CVD 103-HgR vaccine in Mali, West Africa^23^. Healthy adults aged 18–45 years from Bamako received a single oral dose of ≥ 2 × 10^8^ CFU (low-dose) or ≥ 2 × 10^9^ CFU (high-dose) of CVD 103-HgR administered in a buffer cocktail. The original trial included in its design the tertiary objective of assessing the association between *H. pylori* IgG sero-prevalence and the immune response to CVD 103-HgR.

### Definition of the study variables

The dependent variable, vibriocidal seroconversion^23^, was defined as a ≥ fourfold increase in serum vibriocidal antibody titer 14 days post-vaccination compared to the baseline titer.

The main independent variable was *H. pylori* seropositivity at baseline defined as the presence of *H. pylori-*specific IgG antibodies in the pre-vaccination sera (for cutoff value please see the laboratory methods section). *H. pylori-*positive sera were further tested for the presence of IgG antibodies to the cytotoxin-associated gene A (CagA) protein, a marker for virulent *H. pylori* strains. Additional independent variables included serum levels of PGs as a measure of gastric inflammation^24,25^. Serum PGI and PGI are proenzymes of the enzyme pepsin. The PGs are secreted into the gastric lumen and can be found in the serum^26,27^. With increasing severity of *H. pylori* gastritis, serum PGI and PGII levels become elevated, but when atrophic changes occur in the corpus, the PGI level and the PGI:PGII ratio decrease. These markers can predict atrophic gastritis, intestinal metaplasia and gastric cancer^24,25,28,29^. A cutoff value of serum PGI of < 30 μg/L or of a PGI:PGII ratio < 3.0, is used to determine the presence of atrophic gastritis/ gastric atrophy, as recommended by the manufacturer. However, there is no consensus in the literature regarding the best cut-off values^24,25,28–32^. A PGI:PGII ratio of 4 to 6 was proposed to enhance the sensitivity of serum PGs in identifying atrophic gastritis^24,30–32^. The occurrence of atrophic gastritis is low, and increases after age 45 years^33^. Thus, the expected number of persons with atrophic gastritis in this small sample of healthy young adult (ages 18–45, median 22 years) is low. Accordingly, we used the lowest quartiles as cut-off values (73.6 μg/L for PGI and 5.35 for PGI:PGII); these are equivalent to clinically relevant values^24,30–32^.

The following variables were considered as covariates and potential confounders: age in years (analyzed as a continuous variable), and sex. Pre-vaccination vibriocidal antibody titer was also assessed, given our previous observations of an inverse association between baseline levels and the immune response to CVD 103-HgR^6,13,15^.

Since *H. pylori* infection might be a marker of broad exposure to enteric pathogens, we assessed the level of serum IgG antibodies to *Shigella flexneri* 2a lipopolysaccharide as an additional independent variable. This enhances the understanding of the specificity of the association between *H. pylori* infection and oral enteric vaccines.

The categories of *S. flexneri* 2a serum IgG antibody were defined according to the knowledge that *Shigella* is highly endemic in developing countries, already at a very young age^34–36^. Thus, Malian adults were presumed to have been exposed to *Shigella* infections during their life course. Therefore, we compared participants with the highest levels of *S. flexneri* 2a serum IgG antibody (highest quartile [Q4]) to those with lower levels (Q1–Q3).

### Laboratory methods

Sera obtained in the course of the above-mentioned clinical trial^23^ were shipped from Mali to the Center for Vaccine Development and Global Health, University of Maryland School of Medicine, Baltimore, USA and stored at − 20 °C until testing. Vibriocidal antibody levels before and after vaccination were examined as described^23^. Sera were shipped to Tel Aviv University for measurement of *H. pylori* IgG antibody and PGs levels testing. *H. pylori* IgG antibodies were measured using a commercial enzyme linked immunosorbent assay (ELISA) kit (Enzygnost Anti-*Helicobacter pylori* II/IgG kit, Siemens Diagnostics Product GmbH, Marburg, Germany) following the manufacturer's instructions. An optical density (OD) of 0.250 or greater were considered positive. The presence of IgG antibody against recombinant CagA protein was measured in *H. pylori-*positive sera according to an in-house ELISA protocol^18,33^. Concentrations of serum PGI and PGII were quantified by ELISA (Biohit Inc., Helsinki, Finland) following the manufacturer's instructions*.* Serum IgG antibody titers to *S. flexneri* 2a lipopolysaccharide was measured using an in-house ELISA protocol at the laboratory of Prof. Daniel Cohen, Tel Aviv University, Israel^37^ and results were expressed in ELISA units.

### Statistical analysis

Descriptive statistics (medians and interquartile ranges (IQR) for continuous variables, and frequencies and percentages for categorical variables) were used to describe the study sample. Chi square and Fisher Exact tests, where appropriate, were used to examine differences between *H. pylori* IgG seropositive and seronegative vaccinees in categorical variables and Mann–Whitney test for continuous variables (e.g., serum PGs). The Chi square test was also used to examine differences in the percentage of vaccinees who developed vibriocidal antibody seroconversion 14 days following vaccination with CVD 103-HgR, according to *H. pylori* seropositivity and other categorical variables. The difference in the median age between participants with and without seroconversion at day 14 was assessed using the Mann–Whitney test.

A log-binomial model of the generalized linear models^38–40^ was performed to examine the association between *H. pylori* IgG seropositivity and vibriocidal seroconversion, while controlling for potential confounders. The use of the log-binomial model is recommended^38,40^ if the dependent variable is common, as in our study, in which 52/93 (55.9%) of the participants had vibriocidal antibody seroconversion 14 days following immunization with CVD 103-HgR.

The selection of variables to be included in the multivariable analysis was based on our pre-specified main hypothesis of the relation between *H. pylori* seropositivity (the main independent variable) and vibriocidal seroconversion following immunization with CVD 103-HgR, while relying on microbiological, immunological and epidemiological knowledge in the field^21,22^. We adjusted for age given the evidence that this variable might affect the immune response to oral enteric vaccines^21,22^, and that *H. pylori* gastric inflammation and pathology might increase with age^41–44^. Lastly, we considered *S. flexneri I*gG serum antibody as a proxy for exposure enteric infections^21^. Accordingly, we assessed the specificity of the association between *H. pylori* sero-prevalence and vibriocidal seroconversion following immunization with CVD 103 HgR. Relative risk (RR) and 95% confidence intervals (CI) were obtained from this model.

The Mann–Whitney test and box plot were used to assess difference in vibriocidal antibody seroconversion level (with natural logarithm transmutation) according to *H. pylori* seropositivity. In additional analyses we assessed difference in the median baseline level of vibriocidal antibody titer (with natural logarithm transmutation) between participants with and without seroconversion at day 14, using the Mann–Whitney test and box plot. We also performed a multivariable model that included both *H. pylori* seropositivity and baseline vibriocidal antibody level. Statistical significance was set at *p* < 0.05. Data were analysed using IBM SPSS version 25 (Armonk, New York, USA).

### Ethical aspects

The Institutional Review Board (IRB) of the University of Maryland, Baltimore and the local Malian IRB approved the original trial with CVD 103-HgR including the objective of this study that was defined as a tertiary objective. Volunteers were enrolled after providing an informed consent^23^. The ethics committee of Tel Aviv University also approved the protocol of the current study.

We confirm that all methods were performed in accordance with the relevant guidelines and regulations.

## Results

The 93 of 97 participants who had data on both *H. pylori* serostatus and vibriocidal antibody seroconversion 14 days after vaccination with CVD 103-HgR were included in the study. Of these, 39 were males [42%] and the median age was 22 years. The high-dose of CVD 103-HgR (≥ 2 × 10^9^ CFU) was given to 46 participants (49.5%) and the low-dose (≥ 2 × 10^8^ CFU) to 47 subjects (50.5%). *H. pylori* IgG seropositivity was evident in 74 participants (80% [95% CI 70–87%]), of whom 55 (74%) tested positive for CagA IgG antibody. There was no significant difference between *H. pylori* seropositive and seronegative participants with respect to age (*p* = 0.9), sex (*p* = 0.6) or vaccine dose (*p* = 0.2) (Table 1). Serum PGI levels were similar between *H. pylori* seropositive and seronegative vaccinees (median 98.7 µg/L vs. 90.3 µg/L, *p* = 0.3) but a significantly higher level of PGII was found in the *H. pylori* seropositive group (median 14.6 µg/L vs. 10.6 µg/L, *p* = 0.012). The PGI:PGII ratio was significantly lower among *H. pylori* seropositive vaccinees compared to seronegatives: median of 6.6 vs. 8.7, *p* = 0.022 (Table 1).

**Table 1:** Characteristics of vaccinees with CVD 103-HgR oral cholera vaccine by *H. pylori* sero-status.

Variable	Overall, n = 93	*H. pylori* positive, n = 74	*H. pylori* negative, n = 19	*p* value^a^
Age, years (median [IQR])	22.0 (8.5)	22.5 (9.0)	22.0 (9.0)	0.9
Sex, males, n (%)	39 (42%)	32 (43%)	7 (37%)	0.6
High dose of CVD 103-HgR vaccine ≥ 2 × 10^9^ CFU, n (%)	46 (50%)	34 (46%)	12 (63%)	0.18
High level of *S. flexneri 2a* serum IgG antibody, EU^b^, n (%)	24 (26%)	21 (88%)	3 (12%)	0.3
Serum PGI, median (IQR) µg/L	97.9 (48.1)	98.7 (50.4)	90.3 (43.3)	0.2
Serum PGII, median (IQR) µg/L	14.0 (9.6)	14.6 (11.2)	10.6 (6.8)	0.012
PGI: PGII ratio, median (IQR)	6.9 (3.6)	6.6 (3.6)	8.7 (5.4)	0.022

*CFU* colony forming units, *IQR* interquartile range, *PG* pepsinogen.

^a^Fisher Exact test was used for categorical variables and Mann–Whitney for continuous variables.

^b^A high level of *S. flexneri* 2a serum IgG antibody was determined as a level equivalent to the highest quartile (ELISA units ranging between 3,458 and 9,299).

The fold-increase in vibriocidal antibody titer at day 14 compared to the baseline level ranged from 0.02 to 1,024, the value of the 25th percentile was 0.5, the median was 4, and the 75th percentile value was 16. The distribution of the fold increase is presented in Supplementary Table S1. The levels of fold-increase of vibriocidal antibody titer was higher among *H. pylori* positive vs. negative vaccinees (*p* = 0.034 by the Mann–Whitney test, see Supplementary Fig.S1 online).

Four-fold increase in vibriocidal antibody seroconversion 14 days post-vaccination compared to baseline level was evident in 52 participants (56% [95% CI 46–66%]). The median age of participants with vibriocidal antibody seroconversion was 24 years (IQR 9) vs. 21 (IQR 8) among those without seroconversion at day 14, following immunization with CVD 103-HgR (*p* = 0.07 by the Mann–Whitney test). Table 2 describes the bivariate analysis of vibriocidal antibody seroconversion at day 14, after vaccination with CVD 103-HgR, according to other independent variables.

**Table 2:** Vibriocidal antibody seroconversion 14 days after vaccination with a single dose of CVD 103-HgR among Malian adults, according to demographics, vaccine dose, *H. pylori* seropositivity and levels of *S. flexneri* 2a serum antibodies.

Variable	Total	Vibriocidal antibody seroconversion, n (%)	*p* value^a^	RR (95% CI)^b^
**Sex**			0.7	
Males	39	21 (54%)		Reference
Females	54	31 (57%)		1.06 (0.73–1.54)
**Vaccine dose of CVD 103-HgR**			0.9	
Low-dose: ≥ 2 × 10^8^ CFU	47	26 (55%)		Reference
High-dose: ≥ 2 × 10^9^ CFU	46	26 (57%)		1.02 (0.71–1.46)
***H. pylori *IgG seropositivity**			0.004	
Negative	19	5 (26%)		Reference
Positive	74	47 (64%)		2.41 (1.11–5.22)
***S. flexneri 2a* serum IgG antibody, EU**			0.029	
Q1-Q3 (66–3,457)	69	34 (49%)		Reference
Q4 (3,458–9,299)	24	18 (75%)		1.52 (1.09–2.12)

This table describes the bivariate analysis of vibriocidal antibody seroconversion after vaccination with CVD 103-HgR, according to the independent variable.

*CFU* colony forming units, *CI* confidence intervals, *EU* ELISA units, *IgG* immunoglobulin G, *Q* quartile, *RR* relative risk.

^a^The chi square test was used.

^b^Results from an unadjusted generalized linear model with a binomial distribution and log function.

**Figure 1:**
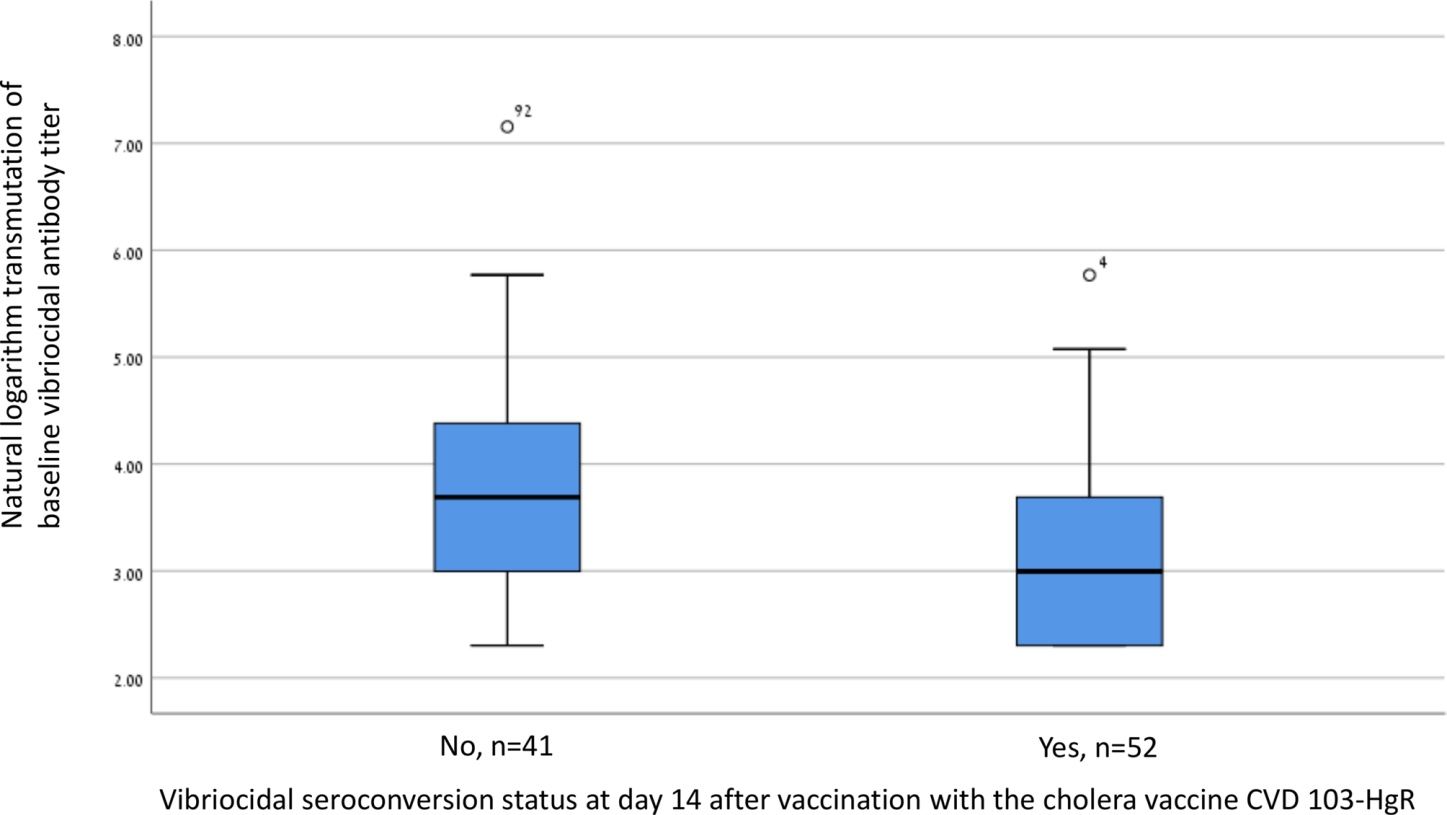
Box plots of baseline vibriocidal antibody titer (natural logarithm transmutation) (Y-axis) according to seroconversion status at day 14 after vaccination with the cholera vaccine CVD 103-HgR (X-axis) among Malian adults. Vibriocidal antibody titer seroconversion was defined as fourfold rise at day 14 after vaccination compared to baseline level. The line in the mid of the box represents the median level, lower bound of the box represents the 25th percentile, the upper bound of the box represents the 75th percentile, the lowest point of the lower whisker represents the minimum and the highest point of the upper represents the maximum. *p* value = 0.001 by Mann–Whitney test.

There was no significant difference in vibriocidal antibody seroconversion according to sex (*p* = 0.7), or vaccine dose (*p* = 0.9). The pre-vaccination vibriocidal antibody level was significantly lower (*p* = 0.001) among persons who seroconverted than among those who did not (Fig. 1).

Vibriocidal antibody seroconversion was significantly higher among *H. pylori* seropositive vaccinees than seronegative ones (64% vs. 26%, *p* = 0.004), and in individuals with high-level (highest quartile) of *S. flexneri* 2a serum IgG anti-LPS antibody than those with lower antibody levels (75% vs. 49%, *p* = 0.029) (Table 2).

Among *H. pylori* seropositive individuals, there was no significant difference between vaccinees with and without vibriocidal antibody seroconversion with respect to CagA IgG seropositivity (70% vs. 82%, *p* = 0.2) or serum PGI, PGII or PGI:PGII levels (Table 3).

**Table 3:** Differences in serum pepsinogens and CagA sero-status between vaccinees with and without vibriocidal antibody seroconversion 14 days after vaccination with CVD 103-HgR among *H. pylori* seropositive persons (n = 74).

Marker	Vibriocidal antibody seroconversion, n = 47	No vibriocidal antibody seroconversion, n = 27	*p* value^a^
**CagA IgG sero-status**			
Negative	14 (30%)	5 (18%)	0.2
Positive	33 (70%)	22 (82%)	
**Serum PGI**^b^			
Q1 (23.6–73.6 µg/L)	11 (23%)	6 (22%)	0.9
Q2–Q4 (73.7–254.6 µg/L)	36 (77%)	21 (78%)	
**Serum PGII**			
Q1 (3.4–13.6 µg/L)	9 (19%)	6 (22%)	0.7
Q2–Q4 (13.7–40.3)	38 (81%)	21 (78%)	
**PGI:PGII ratio**^b^			
Q1 (2.50–5.51)	13 (28%)	7 (26%)	0.8
Q2–Q4 (5.52–16.30)	34 (72%)	20 (74%)	

Data are presented as absolute numbers and percentages in parenthesis.

CagA and serum PGs were measured using baseline sera. Vibriocidal seroconversion was defined as a fourfold rise in vibriocidal titer between baseline and 14 days after vaccination (i.e. using sera from baseline and 14 days after vaccination with CVD 103-HgR).

*CagA* cytotoxin-associated gene A, *IgG* immunoglobulin G, *IQR* interquartile range, *PG* pepsinogen, *Q* quartile.

^a^*p* value was obtained by the chi square test.

^b^The lowest quartile levels of serum PGI and PGI: PGII suggest more severe gastritis.

A multivariable model that adjusted for age (as a continuous variable) and levels of *S. flexneri* 2a serum IgG anti-LPS antibody, showed a significant positive association between *H. pylori* seropositivity and vibriocidal seroconversion: adjusted RR 2.20 (95% CI 1.00–4.80), *p* = 0.049 (Table 4). This model showed no significant association between age and vibriocidal antibody seroconversion (adjusted RR 1.00 [95% CI 0.99–1.03], *p* = 0.4) or levels of *S. flexneri* 2a IgG anti-LPS (adjusted RR 1.32 [95% CI 0.95–1.84), *p* = 0.1).

**Table 4:** The association between *H. pylori* seropositivity and vibriocidal antibody seroconversion 14 days after vaccination with CVD 103-HgR.

	Unadjusted RR^a^ (95% CI)	*P* value^a^	Adjusted RR^b^ (95% CI)	*p* value^b^
***H. pylori IgG serostatus***				
Negative	Reference	0.025	Reference	0.049
Positive	2.41 (1.11–5.22)		2.20 (1.00–4.80)	

*CI* confidence intervals, *IgG* immunoglobulin G, *RR* relative risk.

^a^Results from an unadjusted generalized linear model with a binomial distribution and log function.

^b^Results from a multivariable generalized linear model with a binomial distribution and log function that adjusted for age as a continuous variable and levels of serum *S. flexneri* 2a IgG antibody.

An additional multivariable model included *H. pylori* seropositivity, and baseline vibriocidal antibody level as an additional independent variable (continuous), this did not affect the positive association observed between *H. pylori* and vibriocidal antibody seroconversion (adjusted RR 2.41 [95% CI 1.13–5.12], *p* = 0.022). This model also showed that baseline vibriocidal was inversely related to vibriocidal seroconversion after vaccination with CVD 103-HgR (adjusted RR 0.73 [95% CI 0.60–0.88], *p* = 0.001).

## Discussion

We found a positive association between baseline *H. pylori* IgG antibody seropositivity and vibriocidal antibody seroconversion 14-days following immunization with a single dose of CVD 103-HgR live oral cholera vaccine among Malian adults. This association is reported for the first time in persons from a very low-income country, a setting in which many oral enteric vaccines have shown lower immunogenicity and efficacy compared to populations in high-income countries. The positive association between *H. pylori* infection and vibriocidal antibody seroconversion was independent of age among these adults and of a high level of *S. flexneri* serum IgG anti-LPS antibody, a proxy for exposure to enteric pathogens. Interestingly, the association between *H. pylori* seroprevalence and the immune response to CVD 103-HgR was not affected by baseline vibriocidal antibody level, serum PGs levels, markers of gastric inflammation or infections with *H. pylori* CagA-positive (putatively more virulent) strains.

Age and *S. flexneri* IgG antibodies were related to vibriocidal antibody serconversion in the bivariate analysis only. There was no significant correlation between age and *S. flexneri* IgG antibodies, likely since exposure to *Shigella* infection occurs during childhood in Mali^34–36^.

Findings from the current study confirm our previous observations regarding the involvement of *H. pylori* infection in the immune response to oral enteric vaccines^21,22^. The positive association between baseline *H. pylori* seropositivity and vibriocidal antibody seroconversion following vaccination with CVD 103-HgR among Malian adults is in agreement with findings documented among school-age (5–9 years) Chilean children^21^. In younger Chilean children < 5 years of age, on the other hand, *H. pylori* seroprevalence was inversely related to vibriocidal antibody seroconversion after immunization with CVD 103-HgR^21^. These observations can be explained by the age-related impact of *H. pylori* infection on gastric physiology. *H. pylori* infection is acquired in childhood^45^ and typically most people do not develop symptoms. However, the histopathological severity of gastritis increases with age, even in the absence of symptoms^44^. *H. pylori* antral-predominant chronic gastritis might increase the secretion of gastric acid, resulting in an enhanced gastric acid barrier^20,46^, while *H. pylori* infection and corpus-predominant gastritis might induce gastric hypochlorhydria, which usually occurs in adulthood. Thus, the transit of attenuated oral vaccine strains from the stomach into the small intestine might be enhanced in the presence of hypochlorhydria, and the immune responses to oral vaccines might actually increase. This likely explains our observations in Malian adults.

The finding of a positive association between *H. pylori* seropositivity at baseline and vibriocidal antibody seroconversion following vaccination with CVD 103-HgR in Malian adults corroborates our findings among U.S. adults who were immunized with a live oral typhoid vaccine. In the US adults we observed stronger humoral immune responses following immunization with oral attenuated *Salmonella* Typhi vaccine strain CVD 908-*htrA*^22^ in persons who were *H. pylori* seropositive at baseline and exhibited evidence of advanced gastritis^22^.

In the current study, we did not find an association between serum pepsinogen levels and the immune response to the cholera vaccine CVD 103-HgR among Malian adults. This contrasts with the finding of a positive association between serum pepsinogen level and advanced gastritis among US volunteers^22^. A number of possibilities could explain this discrepancy between the studies, in the associations of serum pepsinogens and the immune response with oral enteric vaccines. One explanation relates to the variation in the epidemiology of *H. pylori* infection and its related diseases; another relates to differences in public health between the US and the sub-Saharan Africa populations^47,48^.

*Salmonella* and *V. cholerae* are both acid sensitive bacteria^49,50^, thus supporting the notion that hypochlorhydria can be the mechanism for the association between *H. pylori* infection and the enhanced immune responses to these vaccines. Further support stems from early studies on cholera disease, showing that hypochlorhydria increases the risk of cholera^49–53^, as well as studies following the discovery of *H. pylori* showing a positive association between *H. pylori* infection and cholera^54–56^ and typhoid fever^57,58^.

The longstanding systemic and local immune responses induced by *H. pylori* infection cannot clear the infection^19,20^. The consequences of persistent *H. pylori* infection may include having an immunomodulatory influence on the immune response to oral enteric vaccines, in particular to cholera and typhoid vaccines. The findings of Mattsson et al.^59^ who assessed the production of antibody responses in the stomach as well as serum antibodies following immunization with an oral B subunit whole-cell cholera vaccine in Swedish volunteers^59^, are consistent with this assumption. Most of their study participants had high levels of vaccine-specific antibody-secreting cells (ASCs) in the duodenum and high serum antibody titers. At the gastric mucosa level, a high frequency of vaccine-specific ASC in the antrum was observed among most *H. pylori-*infected vaccine recipients but not among those who were not infected with *H. pylori*. The *H. pylori-*infected group had substantially higher frequencies of total immunoglobulin A (IgA) ASC in the antrum as compared to the non-infected group^59^. This report with oral killed cholera vaccine is consistent with our findings with live vaccine that pre-existing *H. pylori* infection enhances the immune response to oral cholera vaccines. These observations add evidence to support the hypothesis that although there are deleterious effects of chronic *H. pylori* infection (duodenal ulcers and gastric adenocarcinoma), there may be beneficial effects such as reduced risk of asthma and allergies^46,60^ and less diarrheal disease in some populations^61,62^.

Recent reports showed associations between the gut microbiota and the immune response to oral rotavirus vaccines in Ghana and Pakistan^63–65^, which suggest that modulation of the gut microbiome might yield better immune responses^65^. Collectively, these and our findings support the notion that pre-existing colonization of the gastrointestinal tract with certain bacterial microflora might affect the immunogenicity of oral enteric vaccines.

The relatively small sample size is a potential limitation of our study. Nonetheless, the positive association between *H. pylori* seropositivity and the immune response to CVD 103-HgR was strong and clear, despite the high prevalence of *H. pylori* infection in this cohort, which corroborates prevalence estimates from low-income countries^19,66^. Further studies with larger sample sizes are needed to confirm our findings. Moreover, larger studies with sufficient statistical power are needed to assess possible interactions between *H. pylori* infection and the vaccine dosage on the immune response to the vaccine. The discrepancy between findings from the current study and our previous findings among Chilean children on the association between *H. pylori* sero-prevalence and the immune response to CVD 103-HgR, may stimulate the need for multi-center international studies employing the same protocol to explore differences across populations in this association. Although *H. pylori* seropositivity was measured in baseline sera, prior to immunization, the observational design of our study does not enable causal inference. Since the serological assay that measures *H. pylori* IgG antibody level does not differentiate between past and recent infection, our findings reflect past exposure to *H. pylori* and not necessarily recent infection. However, it is well documented that *H. pylori* infection is mostly acquired in childhood and persists throughout adulthood^19,20^. Moreover, use of a serological assay in this setting in Mali might be the best tool in terms of specificity given reports of potential misclassification due to non-*H. pylori* urease-producing bacteria identified by urea breath test^67^.

Our study has several strengths stemming from both the study design and the novelty of the findings. First, *H. pylori* IgG seropositivity was correlated with serum PGII and PGI:PGII ratio markers of gastric inflammation, rather than just exposure to the infection. Second, the infection was examined in baseline sera, thus establishing a temporal association. Third, the association was specific to *H. pylori* and not related to exposure to other enteric infection such as *S. flexneri*, as demonstrated in the multivariable model. Lastly, the unique positive association between *H. pylori* seroprevalence and the immune response to an oral enteric vaccine (i.e., *V. cholerae* oral vaccine strain CVD 103-HgR) is reported for the first time in sub-Saharan Africa, a setting with frequent cholera outbreaks and a high prevalence of *H. pylori* infection. This increases the generalizability of our findings to other populations in low-income countries.

## Supplementary information

Supplementary file1

## Data Availability

The datasets generated during and/or analyzed during the current study are available from the corresponding author on reasonable request.
