# Supplementary material for: Pre-existing Helicobacter pylori serum IgG enhances the vibriocidal antibody response to CVD 103-HgR live oral cholera vaccine in Malian adults
Source: Sci Rep. 2020 Oct 9;10:16871. doi: 10.1038/s41598-020-71754-9 (PMC7547695; doi:10.1038/s41598-020-71754-9)

Pre-existing *Helicobacter pylori* serum IgG enhances the vibriocidal antibody response to CVD 103-HgR live oral cholera vaccine in Malian adults

Running title: *H. pylori* & cholera vaccine CVD 103-HgR

Khitam Muhsen [a*], Samba O. Sow [b, c], Milagritos D. Tapia [b, c], Fadima C. Haidara [b], Mardi Reymann [c], Valeria Asato [a], Wilbur H. Chen [c], Marcela F. Pasetti [c], Myron M. Levine [c]

[a] Department of Epidemiology and Preventive Medicine, School of Public Health, Sackler Faculty of Medicine, Tel Aviv University, Tel Aviv, Israel.

[b] Centre pour le Développement des Vaccins, Bamako, Mali

[c] Center for Vaccine Development and Global Health, University of Maryland School of Medicine, Baltimore, MD, U.S.A.

***Corresponding author**

Khitam Muhsen, PhD

Department of Epidemiology and Preventive Medicine, School of Public Health, Sackler Faculty of Medicine, Tel Aviv University

Ramat Aviv, Tel Aviv, Israel, 6139001.

Telephone +972-3-6405945, Fax 972-3-6409868.

Email: kmuhsen@tauex.tau.ac.il

Supplementary Table S1: Fold increase in vibriocidal antibody by day 14 post-vaccination with CVD 103-HgR compared to baseline level

Fold-increase	Frequency	Percent
.02	1	1.1
.03	2	2.2
.06	2	2.2
.13	3	3.2
.25	6	6.5
.50	11	11.8
1.00	11	11.8
2.00	5	5.4
4.00	17	18.3
8.00	8	8.6
16.00	12	12.9
32.00	4	4.3
64.00	4	4.3
128.00	3	3.2
256.00	2	2.2
512.00	1	1.1
1024.00	1	1.1
Total	93	100.0

Figure Legend

Supplementary Figure S1: Box plots of the natural logarithm of vibriocidal antibody titer fold-increase, 14 days following immunization with the cholera vaccine CVD 103-HgR (y-axis) among Malian adults, according to *H. pylori* seropositivty (x-axis). The thick line within the box represents the median level, the lower bound of the box represents the 25th percentile, the upper bound of the box represents the 75th percentile, the lowest point of the lower whisker represents the minimum and the highest point of the upper whisker represents the maximum. P value=0.034 by Mann Whitney test for the difference between *H. pylori* positive and negative persons in the natural logarithm of fold-increase in vibriocidal antibody titer 14 after vaccination.

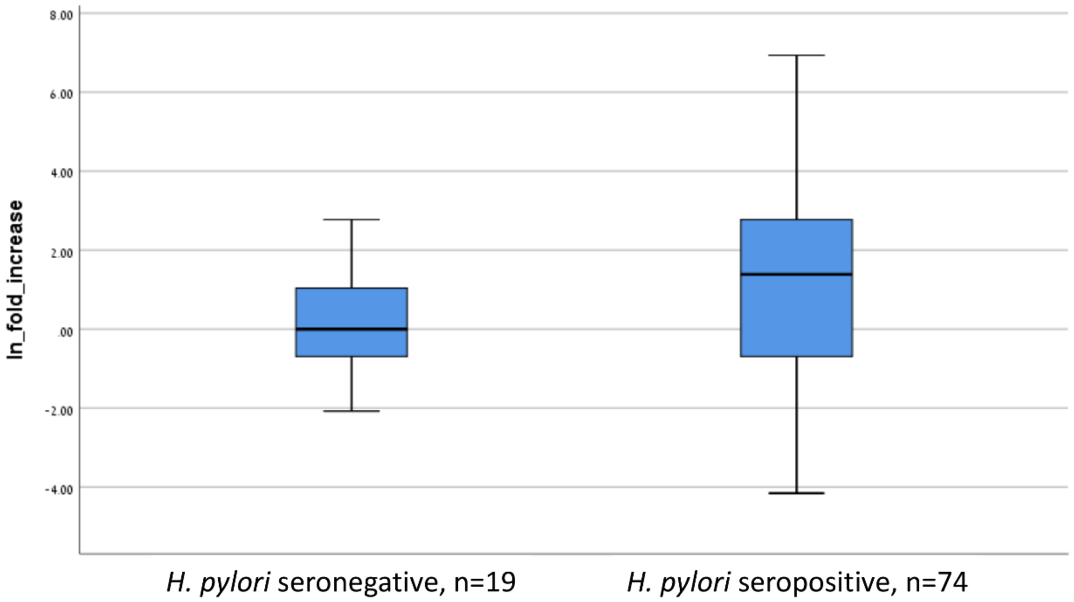

Supplement: Supplementary file 1 — Supplementary file1 [file 41598_2020_71754_MOESM1_ESM.pdf]
